# Venomics of *Trimeresurus (Popeia) nebularis*, the Cameron Highlands Pit Viper from Malaysia: Insights into Venom Proteome, Toxicity and Neutralization of Antivenom

**DOI:** 10.3390/toxins11020095

**Published:** 2019-02-06

**Authors:** Choo Hock Tan, Kae Yi Tan, Tzu Shan Ng, Evan S.H. Quah, Ahmad Khaldun Ismail, Sumana Khomvilai, Visith Sitprija, Nget Hong Tan

**Affiliations:** 1 Department of Pharmacology, Faculty of Medicine, University of Malaya, 50603 Kuala Lumpur, Malaysia;; 2 Department of Molecular Medicine, Faculty of Medicine, University of Malaya, 50603 Kuala Lumpur, Malaysia; kytan_kae@um.edu.my (K.Y.T.); ngtzushan@um.edu.my (T.S.N.); tanngethong@yahoo.com.sg (N.H.T.); 3 School of Biological Sciences, Universiti Sains Malaysia, 11800 Minden, Penang, Malaysia; evanquah@yahoo.com; 4 Department of Emergency Medicine, Universiti Kebangsaan Malaysia Medical Centre, 56000 Kuala Lumpur, Malaysia; khaldun_ismail@yahoo.com; 5 Thai Red Cross Society, Queen Saovabha Memorial Institute, Bangkok 10330, Thailand; sumanaredcross@gmail.com (S.K.); visithstprj@yahoo.com (V.S.)

**Keywords:** *Trimeresurus nebularis*, *Popeia nebularis*, venom proteome, envenomation, proteomics, antivenom, neutralization

## Abstract

*Trimeresurus nebularis* is a montane pit viper that causes bites and envenomation to various communities in the central highland region of Malaysia, in particular Cameron’s Highlands. To unravel the venom composition of this species, the venom proteins were digested by trypsin and subjected to nano-liquid chromatography-tandem mass spectrometry (LC-MS/MS) for proteomic profiling. Snake venom metalloproteinases (SVMP) dominated the venom proteome by 48.42% of total venom proteins, with a characteristic distribution of P-III: P-II classes in a ratio of 2:1, while P-I class was undetected. Snaclecs constituted the second most venomous protein family (19.43%), followed by snake venom serine proteases (SVSP, 14.27%), phospholipases A_2_ (5.40%), disintegrins (5.26%) and minor proteins including cysteine-rich secretory proteins, L-amino acid oxidases, phosphodiesterases, 5′-nucleotidases. The venomic profile correlates with local (painful progressive edema) and systemic (hemorrhage, coagulopathy, thrombocytopenia) manifestation of *T. nebularis* envenoming. As specific antivenom is unavailable for *T. nebularis*, the hetero-specific Thai Green Pit viper Monovalent Antivenom (GPVAV) was examined for immunological cross-reactivity. GPVAV exhibited good immunoreactivity to *T. nebularis* venom and the antivenom effectively cross-neutralized the hemotoxic and lethal effects of *T. nebularis* (lethality neutralizing potency = 1.6 mg venom per mL antivenom). The findings supported GPVAV use in treating *T. nebularis* envenoming.

## 1. Introduction

A large number of the Asian or oriental pit vipers (Reptilia: Serpentes: Viperidae: Crotalinae), in particular those described as having “lance-headed appearance,” were in the past considered congeneric and placed within *Trimeresurus sensu lato* (*s.l.*). Multiple systematic revisions took place over the years on *Trimeresurus* complex with various genera, subgenera and species being erected or collapsed, overwhelming the field with a continuous taxonomic flux [1,2,3]. The exercise has led to at least four genera commonly recognized today for these Asiatic pit vipers: *Trimeresurus sensu stricto* (*s.s.*), *Ovophis*, *Protobothrops* and *Tropidolaemus* (http://reptile-database.reptarium.cz/) [4,5,6]. The *Trimeresurus s.s.* retains the highest number of species, comprising a diverse assemblage of more than 30 known pit vipers [7]. Taxonomic advancements have improved knowledge on field identification and biogeographical distribution of the various species therein [1,2,8]. This is significant to the toxinologist community, as in snake envenomation species identity is crucial for accurate diagnosis and treatment [9]. Extensive biomedical studies have shown that venom compositions can vary greatly between and even within species, and the venom variation usually correlates with differences in venom toxicity and clinical manifestation of snakebite envenoming [10,11,12,13]. More importantly, venom variation is often accompanied by antigenic differences that result in discrepancy of antivenom effectiveness [14,15].

**Figure 1:**
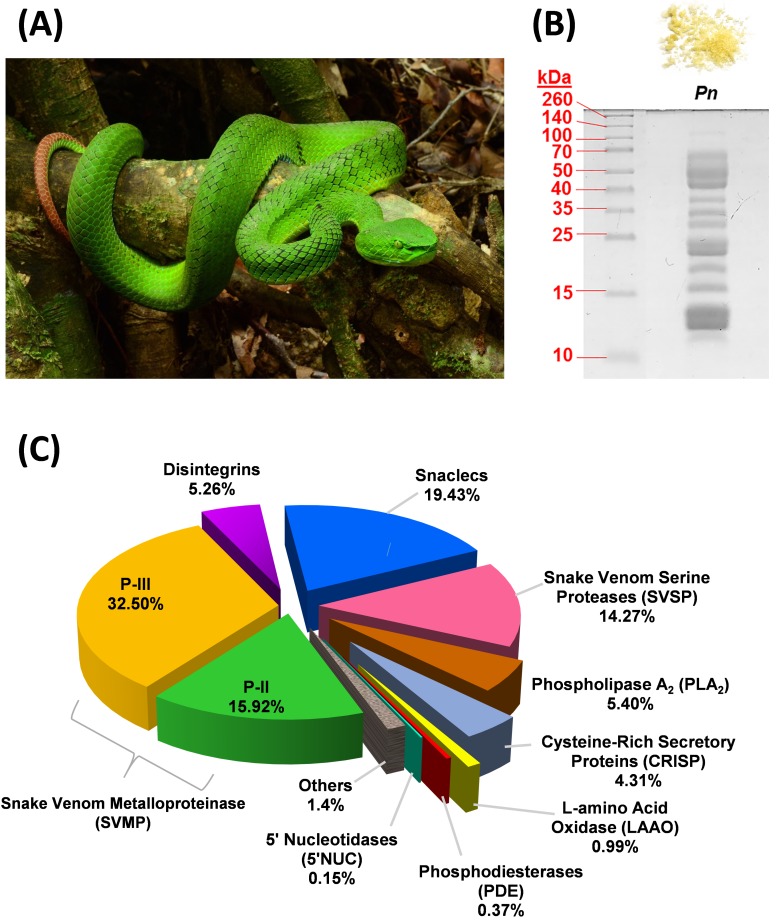
Venomics of *Trimeresurus (Popeia) nebularis* from Malaysia. (**A**) An adult *T. nebularis* perching on a tree branch. Both sexes of this species are “inornata” meaning “unadorned,” lacking ventrolateral stripes. (**B**) 15% SDS-PAGE of *T. nebularis* venom (10 µg) under reducing conditions. Upper panel: lyophilized venom powder with yellow coloration. (**C**) Proteome of *T. nebularis* venom, percentages indicate the relative abundances (% by total venom proteins) of protein family.

Hence, the use of ‘congeneric’ antivenom in cross-neutralizing hetero-specific snake venoms is challenging as the effectiveness of antivenom cannot be simply extrapolated based on the congeneric status of the envenoming species. In Southeast Asia, this medical concern is highly relevant to envenoming by *Trimeresurus* species in view of the diversity and wide distribution of the genus [16]. Among *Trimeresurus* pit vipers, there are endemic species that occupy particular ecological niches, for instance, the Cameron Highlands pit viper—a unique species endemic to the central highland regions of Peninsular Malaysia—commonly received as *Popeia nebularis* in allusion to the cloudy montane rainforests or cloud forests it inhabits (*nebularis*: Latin, “from the clouds”) [17]. The body color of *P. nebularis* is intense green above with a slight bluish tinge but it lacks the ornamentation of brick-red ventrolateral stripes typically present in the adult males of sexually dimorphic members of *Popeia* and that both sexes show reduction of the white lateral stripes—hence, the specific epithet of “inornata” in its junior synonym, *Popeia inornata* [16] (Figure 1A). Currently, *Popeia* has sunken into a subgenus following the re-assignment of nucleo-species to the nominal genus in the *Trimeresurus* systematics [2]. Like most of the *Trimeresurus* species, this species is nocturnal and its preys presumably consist of birds and small mammals, hence some similarities in venom composition may be shared within the *Trimeresurus* complex [18]. The variation in *T. nebularis* venom antigenicity and antivenom neutralization, however, remains to be investigated.

*Trimeresurus nebularis* is restricted to elevations above 1000 m in the Cameron Highlands at the central part of the Titiwangsa Range which forms the mountainous spine of Peninsular Malaysia (type locality: Gunung Brinchang) [16,17,18]. Its occurrence has also been found in Fraser’s Hill and Genting Highlands in the northern part of the Pahang State (Evan SH Quah, pers.com.; http://reptile-database.reptarium.cz/). The distribution of *T. nebularis* causes endemic problem of snakebite envenomation in the montane area of central Malaysia, notably in Cameron Highlands where agricultural activities and eco-tourism are common [19]. Although formal epidemiological report is lacking, hospital records and data collected by the Remote Envenomation Consultancy Service team (RECS, Malaysia) revealed that *T. nebularis* bite is one of the leading causes of envenoming in the affected area, resulting in hemorrhagic syndrome and coagulopathy [20]. As there is no species-specific antivenom available for *T. nebularis*, the hetero-specific Thai Green Pit Viper Antivenom (GPVAV, raised against the venom of *Trimeresurus albolaris*, or *Cryptelytrops albolabris*) has been used empirically in recent years to treat *T. nebularis* envenoming and this approach has been anecdotally reported to be effective based on unpublished clinical observation from the Remote Envenomation Consultancy Service team in the country [19]. The effectiveness of GPVAV is likely attributed to the sharing of venom antigenicity between the two species which are phylogenetically related although they do not share the same habitat and ecological niche [16,18]. In fact, *T. albolabris* has not been known to distribute in Peninsular Malaysia [21]. The hypothesis requires validation of the cross-reactivity and cross-neutralization efficacy of GPVAV against *T. nebularis* venom. Further, the venom composition of this reclassified species should also be elucidated for toxicity correlation and understanding of pathophysiology. Hence, the present study aimed to investigate the proteome and toxicity of *T. nebularis* venom. The immunoreactivity of regional antivenoms toward the venom proteins and the cross-neutralization capacity of GPVAV were also examined for the optimization of antivenom treatment in the country.

## 2. Results and Discussion

### 2.1. Proteome of Trimeresurus nebularis Venom

Sodium dodecyl sulfate-polyacrylamide gel electrophoresis (SDS-PAGE) of *T. nebularis* venom under reducing conditions showed a heterogeneous, complex mixture consisting of proteins with wide ranging molecular weights (Figure 1B). Nano-liquid chromatography-tandem mass spectrometry (LC-MS/MS) of the venom identified a total of 44 proteins in the venom, in which 40 proteins were clustered into 9 different toxin families, namely snake venom metalloproteinase (SVMP, 48.42% of total venom proteins), snake venom C-type lectin/lectin-like protein (snaclec, 19.43%), snake venom serine protease (SVSP, 14.3%), phospholipase A_2_ (PLA_2_, 5.4%), disintegrin (5.26%), cysteine-rich secretory protein (CRISP, 4.31%), L-amino acid oxidase (LAAO, 0.99%), phosphodiesterase (PDE, 0.37%) and 5′-nucleotidase (5′NUC, 0.15%) (Figure 1C). Approximately 1.4% of the venom proteins were consisted of cellular and non-toxin proteins, labeled as “others.” Table 1 shows the details of families, subtypes and relative abundances of proteins in *T. nebularis* venom proteome. The venom proteome was assembled by analysis of LC-MS/MS data as provided in Table S1.

SVMP formed the main bulk of *T. nebularis* venom proteome (close to 50% of total venom proteins). A total of 19 SVMP proteins were identified; among these, 11 proteins were of P-III class (32.50% of total venom proteins) and 8 proteins were of P-II class (15.92%). SVMP are typical venom proteins present substantially in viperid venoms, known for causing hemorrhage and hemostatic derangement: SVMP proteins damage the collagenous basement membrane and extracellular matrix, thereby weakening the vascular barriers and facilitating blood loss from the vasculature of prey or humans envenomed [22]. The distribution of SVMP subtypes in *T. nebularis* venom revealed a dominating trend of class P-III over P-II (at a ratio of 2:1), while P-I was undetected in this study. The dominance of P-III in the SVMP subproteome of *T. nebularis* venom is consistent with the quantitative venom proteome reported for *Viridovipera stejnegeri* (Taiwan), an arboreal species belongs to *Trimeresurus* complex, in which P-III class was shown to be the main SVMP component [23]. The feature has also been observed in the venom proteomes of other land-dwelling pit vipers in Asia, including short-tailed mamushi (*Gloydius brevicaudus*, China) whose venom showed a richer content of P-III than P-II without P-I [24] and *G. intermedius* whose venom contained only P-III class of SVMP [25]. Similarly, P-III class of SVMP predominates in the venom proteome of Wagler’s pit viper (*Tropidolaemus wagleri*, Malaysia), albeit the overall quantitative abundance of SVMP in the proteome is very low (~1.72%) [26], unlike those of the *Trimeresurus*, *Protobothrops* and *Gloydius* complexes (SVMP content is typically >20% or as high as ~50% of total venom proteins as in this study) [13,23,24,27]. Meanwhile, a recent proteomic study indicates that the SVMP (31.24%) in *Protobothrops flavoviridis* venom were mainly P-II subtypes [13]. The unique distribution pattern of SVMP classes (P-III:P-II:P-I = 2:1:0) observed in *T. nebularis* venom, however, contrasts with the lack of P-III components shown in the quantitative venom proteomes of *Hypnale hypnale* (hump-nosed pit viper, Sri Lanka) [28] and *Calloselasma rhodostoma* (Malayan pit viper, Malaysia) [29]. The finding suggests a potential dichotomous distribution of SVMP classes between the P-III predominant group (arboreal pit vipers of *Trimeresurus* complex, land pit vipers of *Gloydius* sp.) and P-I predominant group (basal Asiatic pit vipers in particular *H. hypnale* and *C. rhodostoma*). Structurally, the metalloproteinase domain is conserved in all SVMP classes, while losses of other domains for example, cysteine-rich and disintegrin components from P-III resulted in the emergence of P-I and P-II, thus promoting gene minimization and focused activity of the enzyme [30]. Functionally, the metalloproteinase domain is responsible for hemorrhagic effect and the enzyme proteolytic activity could contribute to fibrinogenolytic and procoagulant effects of the venom [22,31]. Further, P-II and P-III SVMPs may also exhibit platelet aggregation inhibiting activity because of the presence of a disintegrin domain in its C-terminus [32,33]. The dominance of SVMP in *T. nebularis* venom proteome corroborate the clinical effect of *T. nebularis* envenoming, which include bleeding from the bite site, coagulopathy marked by prolonged prothrombin time and in some, thrombocytopenia which further aggravates uncontrolled bleeding [19].

Snaclecs, that is, C-type lectins or lectin-like proteins derived from snake venoms, constitute the second most abundant proteins in *T. nebularis* venom proteome (~20% of total venom proteins). The majority of the subtypes (4 out of 6 snaclec proteins identified) were highly homologous to C-type lectins of *Trimeresurus (Viridovipera) stejnegeri*, a pit viper species of the *Trimeresurus* complex. The snaclec abundance in *T. nebularis* venom proteome is considerably higher compared to *T. stejnegeri* (1.5%), *P. flavoviridis* (2.78%) and *Trimeresurus/Cryptelytrops purpureomaculatus* (2.44%) reported previously [13,23,27]. The snaclecs identified are mostly platelet aggregation activators which act by cross-linking platelet membrane GP Ib-IX-V receptor complex, thereby inducing platelet aggregation [34]. The aggregation of platelet leads to the “consumption” of normal, functional platelets and thus resulting in thrombocytopenia. The high abundance of snaclec proteins in *T. nebularis* venom correlates with the thrombocytopenic picture observed clinically in *T. nebularis* envenoming (Remote Envenomation Consultancy Service, pers. com.). On a side note, thrombocytopenia is also a common clinical feature of Malayan pit viper (*C. rhodostoma*) and hundred-pace pit viper (*Deinagkistrodon acutus*) envenomings and the effect has been correlated to the abundant snaclecs or C-type lectins found in the venoms (20–40%) [29,35]. In contrast, *T. stejnegeri* and *Protobothrops mucrosquamatus* envenomings rarely cause thrombocytopenia [36] and this has been attributed to the low abundance of C-type lectins in *T. stejnegeri* and *P. mucrosquamatus* venoms (1.5% and 3.9%, respectively) [23]. The composition difference of snaclec proteins among different pit viper venoms provides a scientific basis for distinguishing thrombocytopenic *D. acutus* bite from the hemorrhagic *T. stejnegeri* and *P. mucrosquamatus* bites by clinicians in Taiwan [35,36]. Nonetheless, in many Southeast Asian countries including Malaysia, this syndromic divergence is not clear-cut and non-specific, as many *Trimeresurus* sp. including *T. nebularis*, like *C. rhodostoma*, do cause thrombocytopenia besides hemorrhage in envenoming [20,37]. The overall hemotoxic syndrome is a constellation of effects induced by the abundant SVMP hemorrhagins, platelet-disrupting snaclecs and other coagulopathic toxins (such a SVSP and disintegrins discussed below) in their venoms.

Snake venom serine proteases (SVSP) are primarily found in viperid venoms that exhibit procoagulant properties [38,39]. A total of five SVSP proteoforms, constituting 14.27% of total venom proteins were identified in *T. nebularis* venom. The SVSP proteoforms include fibrinogenase and thrombin-like enzyme such as ancrod, a potent defibrinating agent developed from *C. rhodostoma* venom [40]. Thrombin-like enzymes act via cleavage of fibrinogen chains, leading to the formation of fibrin microclots which are friable and rapidly dissolved [37]. The activation of fibrinogen is pathological as it leads to consumptive coagulopathy *in vivo*, a common complication reported in *T. nebularis* envenoming (Remote Envenomation Consultancy Service, pers. com.). Like ancrod, the procoagulant SVSPs are toxins with therapeutic potential for further investigation into their *in vivo* anticoagulant activities and hence anti-thrombotic drug development.

Besides, two disintegrins, constituting approximately 5% of total venom proteins, were identified in the *T. nebularis* venom proteome. The disintegrins were matched by sequence homology to trigramin and ussuristatin which are platelet glycoprotein IIb-IIIa protein antagonists that inhibit platelet aggregation [41,42]. The disintegrin activity may further interrupt normal platelet function and this leads to worsening of hemorrhagic syndrome affecting the envenomed patient.

There were two PLA_2_ (~5% of total venom proteins) detected in *T. nebularis* venom proteome. The low abundance of PLA_2_ is similar to that reported in the venom proteomes of *C. rhodostoma* and *D. acutus* (<10%) [29,35] but differs markedly from that reported for the arboreal *T. stejnegeri* (~20%), *P. mucrosquamatus* (~25%) [23], *P. flavoviridis* (~55%) [13] and *T. purpureomaculatus* (33.01%) [27]. One of the PLA_2_ proteoforms identified in *T. nebularis* venom proteome is homologous to the Lys-49 basic PLA_2_ of *P. mucrosquamatus* (P22640) known for edema-inducing property [43], supporting the inflammatory feature of progressive painful edema in local envenoming by the pit viper. The other unique PLA_2_ subtype was annotated by a PLA_2_ gene sequence (A0A0H3U232) from *Trimeresurus sabahi*; the function of this PLA_2_ subtype remains unknown. Meanwhile, other proteins detected in *T. nebularis* venom proteome include cysteine-rich secretory proteins (CRISP, ~4%) and minor enzymes (L-amino acid oxidase, phosphodiesterase, 5′-nucleotidase, with abundance ≤1% each). These proteins are not known to play essential roles in the pathophysiology of snakebite envenoming but they probably contribute to ancillary functions that facilitate prey immobilization and digestive process [37,44,45].

The present study employed the shotgun proteomic approach which, theoretically, allows unbiased capturing of digested peptides present in the sample, hence providing a broader detection range for proteins with low abundance and extreme isoelectrical point (pI) or molecular weight [46]. In addition, the availability of peptide intensity and spectral count information in shotgun proteomics allows relative quantification of proteins present in the sample [47,48]. Nevertheless, the information on protein subtypes and protein separation could be limited, unlike protein decomplexation strategy that integrates reverse-phase HPLC [49]. Besides, the estimation of protein abundance in shotgun proteomics relies on the availability of a good MS/MS spectral matching reference database, as any paucity in homologous sequence database can lead to missing data [47]. In addition, the current work successfully detected the venom peptide disintegrins but no metalloproteinase inhibitor or bradykinin-potentiating peptides were detected. As the latter two peptide components were relatively less reported in snake venoms, it could be possible that these peptides were not present in the venom or only expressed at a trace amount that is below the detection limit of mass spectrometry. Experimentations with improved peptide resolution and detection capability should be explored in the future.

### 2.2. Immunological Profiling of T. nebularis Venom

**Figure 2:**
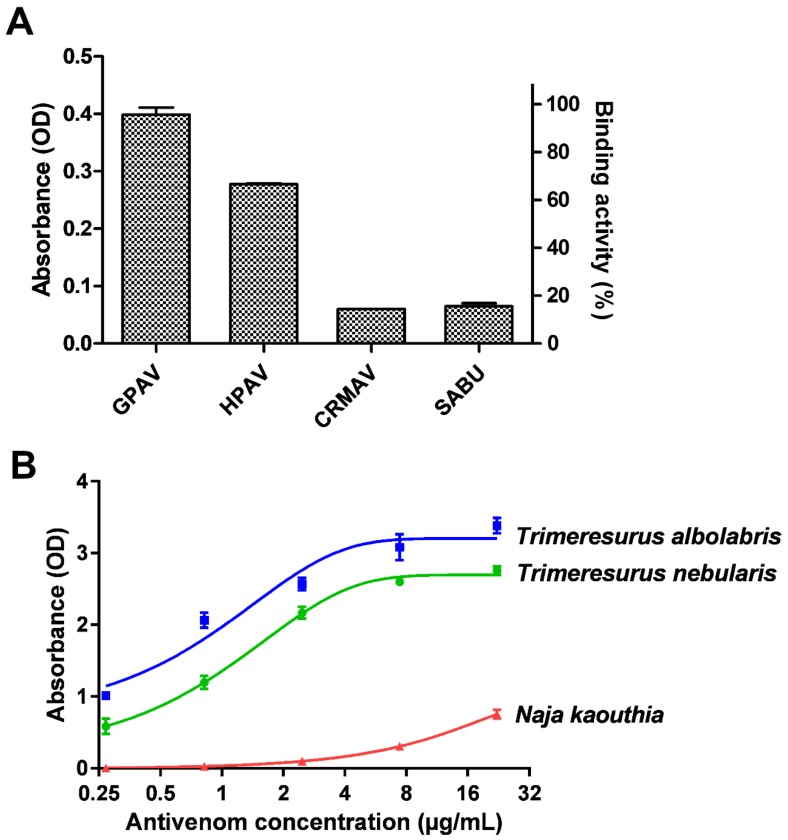
Immunological profiling of *Trimresurus nebularis* venom (Malaysia). (**A**) Cross-reactivity of GPVAV, HPAV, CRMAV and SABU toward *Trimeresurus nebularis* venom. Green Pit Viper Antivenom (Thailand); HPAV: Hemato Polyvalent Antivenom (Thailand); CRMAV: *Calloselasma rhodostoma* Monovalent Antivenom (Thailand); SABU: Serum Anti Bisa Ular (Anti-snake venom, Indonesia). (**B**) Concentration-response curve of GPVAV immunological binding to *T. nebularis* venom protein antigens. *Trimeresurus albolabris* venom (Thailand): positive control; *Naja kaouthia* venom (Thailand): negative control. Values were means ± SEM of triplicate experiments.

In antivenom immunoreactivity study, our preliminary findings revealed that GPVAV exhibited the highest cross-reactivity (followed by Hemato Polyvalent Antivenom, HPAV) toward *T. nebularis* venom antigens, exceeding the immunoreactivities shown by *Calloselasma rhodostoma* Monovalent Antivenom (CRMAV, the Malayan pit viper antivenom) and Serum Anti Bisa Ular (SABU, the Indonesian trivalent antivenom) by at least 4 folds (Figure 2A). GPVAV showed a dose-dependent increase in immunological binding activity to *T. nebularis* venom, with the median effective concentration, EC_50_ determined as 1.2 µg/mL. The binding potency of GPVAV to *T. nebularis* venom was slightly lower than binding to the homologous Thai *T. albolabris* venom (EC_50_ = 0.7 µg/mL). The ability of GPVAV to cross-react with *T. nebularis* venom proteins indicates that common protein antigenicity is shared between the venoms of *T. albolaris* (species from which GPVAV was raised against) and *T. nebularis*. By the current systematics both species were divided into two different subgenera: *Trimeresurus* subgenus for *T. albolabris* and *Popeia* subgenus for *T. nebularis* [2]. The finding supports that the venom protein antigenicity is well conserved between the two different subgenera of *Trimeresurus* species, despite their morphological and ecological differences in particular related to habitat and geographical distribution [7,8,17]. On the other hand, *Trimeresurus* sp. are more distantly separated from *C. rhodostoma* in the phylogenetics of Asian pit vipers [50], hence the more variable venom antigenicity and a much lower cross-reactivity level of CRMAV and SABU which were raised against *C. rhodostoma* venom.

### 2.3. Cross-Neutralization of T. nebularis Venom Toxicity

*Trimeresurus nebularis* venom was procoagulant in human citrated plasma and it showed local hemorrhagic effect in mice (Table 2), consistent with the findings of abundant SVMPs and SVSPs in the venom proteome. The venom has an intravenous median lethal dose (LD_50_) of 2.0 µg/g in mice, a value considerably higher (less lethal) than the reported value for the deadly Malayan pit viper (*Calloselasma rhodostoma*, 1.48 µg/g) [51]. The LD_50_ is closer to values reported for a number of other viper and pit viper venoms, for example, *Trimeresurus stejnegeri* (~2 ug/g) [23], *Protobothrops flavovi*ridis (2.5 µg/g) [52], *Echis carinatus sochureki* (2.08 µg/g) and *Bothrops erythromelas* (1.80 µg/g) [53]. The *Trimeresurus* complex is diverse and within the species complex, the venom LD_50_ values commonly range between 0.5–2.0 µg/g [37,54]. As the lethality of venom is a combined effect of various toxins, the LD_50_ values could vary substantially between species depending on the venom protein composition. In this study, although *T. nebularis* venom has a relatively high LD_50_, it should be noted that a huge amount of venom could be potentially injected by an adult *T. nebularis* in each bite (~10–30 mg per milking, author’s observation). Considering that the pharmacokinetics of pit viper venom proteins typically depicts a continuous absorption phase and a prolonged half-life of days [55], the hemotoxic effect (including coagulopathy and hemorrhage) of *T. nebularis* venom may develop insidiously and prolong over hours or even days, culminating in shock, organ failure and ultimately death if proper treatment is not available.

Antivenom is the only definitive treatment for snakebite envenoming. In the absence of a species-specific antivenom, a paraspecific antivenom may be considered for possible cross-neutralizing effect [56,57]. Based on the immunoreactivity findings in the present work, the Thai Green Pit Viper Antivenom (GPVAV) was further explored for its capability to cross-neutralize the coagulopathic and hemorrhagic as well as lethal effects of the venom. GPVAV effectively cross-neutralized the toxicity tested and the efficacy parameters were shown in Table 2. The cross-neutralization of GPVAV corroborated the effective immunological binding activity of GPVAV to *T. neubularis* venom (Figure 2B) and indicated that the principal hemotoxins in both *T. nebularis* and *T. albolabris* shared substantial similarities in protein antigenicity. To further examine the specific binding activity of antivenom to different toxin proteins, it is recommended that the antivenomic approach is employed in the future. This can be achieved by integrating a protein decomplexation method for example, reverse-phase high performance liquid chromatography of the crude venom, followed by immunoblotting or affinity chromatograph of the eluted protein fractions.

Together, the immunological binding of GPVAV to *T. nebularis* venom and the functional neutralization of the venom hemorrhagic, coagulopathic as well as lethal effects attested the efficacy of GPVAV in treating envenoming by this species. The findings were also in agreement with the previously reported effectiveness of GPVAV in cross-neutralizing venoms from a number of Indonesian pit vipers (*Trimeresurus* sp.) [54]. The potency values of GPVAV (interpreted as the amount of venom completely neutralized by a unit volume or a unit mass of antivenom) were considerably high (P = 1.6 mg venom per mL antivenom or normalized potency, n-P = 79.2 mg venom per g antivenom). It is theoretically possible to extrapolate that in *T. nebularis* bite, a total of 30 mg of venom injected and systematically absorbed can be completely neutralized by 20–30 mL of GPVAV (equivalent to 2–3 vials of antivenom), which appears to concur with the initial dose of GPVAV recommended in clinical guidelines [19,20]. Nonetheless, from the practical standpoint, the dosing of GPVAV should be determined and optimized clinically, as the neutralization effect of antivenom is modulated by complex pharmacokinetics of both venom and antivenom proteins in *in vivo* system. Unmatched venom and antivenom disposition can lead to resurgence of venom levels and re-envenoming phenomenon in the patient who may have seemingly recovered following initial antivenom treatment [58,59]. Close monitoring of patient’s progress is hence warranted throughout and after antivenom treatment.

## 3. Conclusions

Envenoming by *T. nebularis* affects agricultural populations in the central highland region of Malaysia, in particular Cameron’s Highlands based on the record of Remote Envenoming Consultancy Service and clinical guideline available in the country [19]. Despite being an endemic issue, the medical burden associated is detrimental to the well-being of the victim, his or her family and to national economic development. In this study, the quantitative proteome and toxicity study of *T. nebularis* venom elucidated the pathophysiology of the envenoming caused by this species. The study further showed that the venoms of *T. nebularis* (Malaysia) and *T. albolabris* (Thailand) shared common protein epitopes. It also validated that the hetero-specific GPVAV raised against Thai *T. albolabris* is effective in cross-neutralizing the hemotoxic and lethal effects of *T. nebularis* venom. The findings are in line with clinical observation where empirical use of GPVAV successfully treated *T. nebularis* envenoming (Ismail A.K., pers. com.). It indicates that the paraspecific use of GPVAV is a reasonable practice in this context whilst awaiting clinical trial.

## 4. Materials and Methods

### 4.1. Venoms and Antivenoms

The venom of *T. nebularis* (Malaysia) was a pooled sample from eight adult snakes, comprising 4 male and 4 female wild-caught specimens from Cameron Highlands. Milking was done by author C.H.T. and the venom was subsequently freeze-dried for stability during storage. The venom of Thai *T. albolabris* was supplied by Queen Saovabha Memorial Institute, Bangkok, Thailand. Thai *Naja kaouthia* venom was a gift from Professor Kavi Ratanabanangkoon of Mahidol University, Thailand. All venom samples were kept in lyophilized state at −20 °C until use.

The main antivenom used in this study, Green Pit Viper Monovalent Antivenom (GPAV, batch no. TA00812) contained purified F(ab)’_2_ derived from serum of horses hyperimmunized against Thai *Trimeresurus albolabris* (white-lipped pit viper) venom and produced by Queen Saovabha Memorial Institute (QSMI), Bangkok, Thailand. Other antivenoms used only in immunological binding study were Hemato Polyvalent Snake Antivenom (HPAV, batch no. HP00216), Malayan pit viper (*C. rhodostoma*) Monovalent Snake Antivenom (CRMAV, batch no. CR00210) and Serum Anti Bisa Ular (SABU or Biosave^®^, batch no. 4701314). HPAV was derived from serum of horses hyperimmunized against the venoms of *C. rhodostoma*, *T. albolabris* and *Daboia siamensis* (Russell’s viper), CRMAV was derived from serum of horses hyperimmunized against the Thai *C. rhodostoma* venom. Both HPAV and CRMAV were produced by QSMI. SABU, manufactured by BioFarma Pharmaceuticals, Bandung, Indonesia, was derived from the sera of horses hyperimmunized against three venoms of Indonesian origin from *N. sputatrix* (Javan spitting cobra), *Bungarus fasciatus* (banded krait) and *C. rhodostoma*.

### 4.2. Estimation of Antivenom Protein Concentration

Protein concentrations in antivenoms (GPAV, HPAV, CRMAV and SABU) were determined using Thermo Scientific™ Pierce™ BCA (bicinchoninic acid) Protein Assay Kit (Thermo Scientific™ Pierce™, Waltham, MA, USA). The protein concentrations were expressed as means ± SEM of triplicates.

### 4.3. Whole Venom In-Solution Tryptic Digestion and Protein Identification by Tandem Mass Spectrometry (Nano-ESI-LC-MS/MS)

The in-solution digestion of whole venom sample was performed in three technical replicates. Twenty micrograms of the venom was reduced with dithiothreitol, alkylated with iodoacetamide and digested with mass-spectrometry grade trypsin protease as previously described [60]. The trypsin-digested peptides were cleaned up and desalted using Millipore ZipTip^®^ C_18_ Pipette Tips (Merck, Burlington, MA, USA). The peptide eluates were subsequently reconstituted in 7 µL of 0.1% formic acid in water. The separation of peptides were carried out on a 1260 Infinity Nanoflow LC system (Agilent, Santa Clara, CA, USA) coupled to Accurate-Mass Q-TOF 6550 series equipped with a nanoelectrospray ionization source. The peptides were resolved by HPLC Large-Capacity Chip Column Zorbax 300-SB-C18 (160 nl enrichment column, 75 µm × 150 mm analytical column and 5 µm particles) (Agilent, Santa Clara, CA, USA). The injection volume was 1 µL per sample. A flow rate of 0.4 µL/min was applied along with a linear gradient of 5-70% of Solvent B (0.1% formic acid in 100% acetonitrile). The drying gas flow rate was 11 L/min at 290 °C. The fragmentor voltage was set to 175 V and the capillary voltage was 1800 V. The ion mass spectra were acquired in a tandem mass spectrometry (MS/MS) mode using Mass Hunter acquisition software (Agilent, Santa Clara, CA, USA), where a MS scan range of 200–3000 *m*/*z* and a MS/MS scan range of 50–3200 *m*/*z* were chosen. Data with a MH+ (positive ion) mass range between 50 and 3200 Da were extracted and analyzed by Agilent Spectrum Mill MS Proteomics Workbench software packages version B.04.00 (Agilent Technologies, Santa Clara, CA, USA). The peptide masses were searched against a non-redundant database from NCBI (taxonomy: Serpentes, taxid: 8570) and an in-house database of venom-gland transcripts obtained through *de novo* transcriptomic method [61,62]. Carbamidomethylation was specified as a fixed modification and oxidized methionine as a variable modification. Proteins identified were validated with the following filters: protein score >20, peptide score >10, scored peak intensity (SPI) >70% and false discovery rate (FDR) <1%. The abundance of individual venom toxin was estimated based on its mean spectral intensity (MSI) relative to the total spectral intensity of all proteins identified [48].

The mass spectrometry acquisition data peak file was deposited in the ProteomeXchange repository (PX) (http://www.proteomexchange.org/) under Integrated Proteome Resource (iProX) (http://www.iprox.org) with project ID: IPX0001476000 and subproject ID: IPX0001476001.

### 4.4. Sodium Dodecyl Sulphate-Polyacrylamide Gel Electrophoresis (SDS-PAGE)

SDS-polyacrylamide gel electrophoresis (SDS-PAGE) was performed according to the method of Laemmli (1970) [63]. The protein molecular weights were calibrated using the Thermo Scientific™ Spectra™ Multicolor Broad Range Protein Ladder (10−260 kDa). *Trimeresurus nebularis* venom (10 µg) was loaded onto a 15% gel and the electrophoresis was performed under reducing conditions at 80 V for 2.5 h. Proteins were stained with Coomassie Brilliant Blue R-250 for visualization.

### 4.5. Immunological Binding Assay

Screening of immunological reactivity between *T. nebularis* venom antigens (10 ng) and antivenoms (GPVAV, HPAV, CRMAV, SABU at 1:6000 dilution from a stock of 20 mg/mL) were examined with an indirect enzyme-linked immunosorbent assay (ELISA) as previously described [64]. GPVAV which exhibited the highest cross-reactivity was further prepared in serial dilutions (1:300, 1:900, 1:2700, 1:8400; 1:25,200) to investigate its concentration-dependent immunological binding activity to *T. nebularis* venom antigens. In brief, the immunoplate wells were precoated with 10 ng venoms of *T. nebularis*, *T. albolabris* (positive control) and *N. kaouthia* (negative control) at 4 °C overnight. Subsequently, the plate was flicked dry and rinsed four times with phosphate-buffered saline with 0.5% Tween^®^20 (PBST). A hundred microliters of serially diluted antivenom (as above mentioned, diluted from a stock of 20 mg/mL) was added to each antigen-coated well, followed by incubation for 1 h at room temperature. After washing the plate four times with PBST, 100 µL of appropriately diluted horseradish peroxidase-conjugated antihorse-IgG (Jackson ImmunoResearch Inc., West Grove, PA, USA) in PBST (1:10,000) was added to each well and incubated for another hour at room temperature. The excess components were removed by washing four times with PBST. Subsequently, 100 µL of freshly prepared 3,3’,5,5’-tetramethylbenzidine substrate solution was added to each well. The enzymatic reaction took place in the dark for 30 min at room temperature and terminated by adding 50 µL of 12.5% sulphuric acid to each well. Next, the absorbance at 412 nm was read using Tecan Infinite M1000 Pro plate reader (Tecan Laboratories, Männedorf, Switzerland). Values were means ± SEM of triplicate experiments.

### 4.6. Neutralization of T. nebularis Venom Procoagulant Effect

Procoagulant activity of the venom was determined using a modified turbidimetric method [10]. A hundred microliters of citrated human plasma was added to 100 µL of *T. nebularis* venom of various concentrations at 37 °C. The absorbance at 405 nm which signaled plasma clotting was monitored every 30 s over a duration of 30 min. A plot of absorbance versus time (min) was generated, where an initial lag time was followed by a drastic increase in absorbance due to rapid clot formation. The increase in absorbance came to a plateau then. The clotting time was determined as the time when the absorbance became 0.02 U greater than the mean of the first two absorbance measurements. The minimum clotting dose (MCD) was defined as the dose of venom that induced coagulation in 5 min.

In in vitro neutralization study, a venom amount equivalent to 2 MCD was pre-incubated with serially diluted antivenom (GPVAV) at 37 °C for 30 min. This was followed by the addition of 100 µL citrated human plasma and the clotting time of the plasma was determined as described above. The effective dose (ED) and effective ratio (ER) of GPVAV were then determined. ED was defined as the dose of antivenom that prolonged the clotting time of plasma to 3 times that of the control (2 MCD, without antivenom). ER was defined as the amount of venom neutralized per unit volume of antivenom (mg venom/mL antivenom) at which the clotting time of challenge dose was prolonged by 3 times.

### 4.7. Neutralization of T. nebularis Venom Toxicity

The hemorrhagic assay was performed by intradermal injection of venom into the dorsal skin of ICR (Institute of Cancer Research strain) mice (20−25 g, *n* = 3) as previously described [65]. The animals were euthanized by CO_2_ 90 min later and the skins were removed. Minimal hemorrhagic dose (MHD) was defined as the amount of venom that induced a skin hemorrhagic lesion of 10 mm diameter. In neutralization assay, various doses of antivenom (GPVAV) were pre-incubated with a constant amount of venom challenge dose (2 MHD) at 37 °C for 30 min prior to intradermal injection. The neutralization of hemorrhagic effect was expressed as median effective dose (ED_50_), defined as the dose of antivenom in µL at which the venom hemorrhagic activity was reduced by 50%. Median effective ratio (ER_50_) was defined as the amount of venom neutralized per unit volume of antivenom (mg venom/mL antivenom) at which the venom hemorrhagic activity was reduced by 50%.

The median lethal doses (LD_50_) of venom was determined by injection via caudal veins into ICR mice (*n* = 4 per dose, 20–25 g). The survival ratio was rcorded after 24 h. In neutralization, venom and antivenom (GPVAV) were preincubated as described by Tan et al. [56]. A challenge dose at 5 LD_50_ of the venom was dissolved in normal saline and pre-incubated with various dilutions of GPAV at 37 °C for 30 min. The mixture was then injected intravenously and the survival ratio was recorded after 24 h. Antivenom neutralizing capacity was expressed as median effective dose (ED_50_), defined as the unit of reconstituted antivenom that gives 50% survival in the venom-challenged animals. The parameters were determined according to the Probit analysis method of Finney using BioStat 2009 analysis software (AnalystSoft Inc., Vancouver, BC, Canada). Neutralization capacity was also expressed in term of ‘neutralization potency’ (P, defined as the amount of venom neutralized completely by a unit of antivenom) according to the calculation of Morais et al. [66]. The neutralization potency is a parameter that is unaffected by the number of LD_50_ chosen as challenge dose. To standardize the amount of antivenom, the P value of GPVAV was normalized by the antivenom protein concentration. The parameter derived is termed normalized potency (n-P), defined as the amount of venom neutralized per gram of antivenom protein [67]. The venom neutralization study followed the guideline of WHO [68] and the animal experiment was approved by the Institutional Animal Care and Use Committee, University of Malaya (2017-200309/PHAR/R/LJL, date of approval: 9 March 2017).

## Figures and Tables

**Table 1:** Overview of the protein families, subtypes and relative abundances (%) of *Trimeresurus (Popeia) nebularis* venom.

Protein Family/Protein Identity ^a^		Database Accession ^b^	Species ^c^	Relative Abundance ^d^
Snake venom metalloproteinases (SVMP)				48.42%
	***P-II subtypes (1-8)***			***15.92%***
1	Zinc metalloproteinase/disintegrin	Q805F4	*Agkistrodon piscivorus piscivorus*	4.28%
2	Zinc metalloproteinase-disintegrin stejnihagin-B	CL4568.contig1_Cp	*Trimeresurus purpureomaculatus*	3.20%
3	Zinc metalloproteinase-disintegrin stejnitin	P0DM87	*Trimeresurus stejnegeri*	2.60%
4	Zinc metalloproteinase/disintegrin	P0C6E4	*Protobothrops jerdonii*	2.53%
5	Metalloproteinase 3	CL174.contig3_CrT	*Calloselasma rhodostoma*	2.17%
6	metalloproteinase 9	CL92.contig6_Ta	*Trimeresurus albolabris*	0.74%
7	P-II metalloprotease	T2HRS1	*Protobothrops flavoviridis*	0.25%
8	Zinc metalloproteinase homolog-disintegrin albolatin	P0C6B6	*Trimeresurus albolabris*	0.15%
	***P-III subtypes (9-19)***			***32.50%***
9	metalloproteinase isoform 1	CL83.contig1_Ta	*Trimeresurus albolabris*	6.63%
10	Zinc metalloproteinase-disintegrin ACLD	CL92.contig4_Ta	*Trimeresurus albolabris*	5.94%
11	Zinc metalloproteinase-disintegrin TSV-DM	CL83.contig2_Ta	*Trimeresurus albolabris*	3.06%
12	Zinc metalloproteinase-disintegrin-like stejnihagin-B	Q3HTN2	*Trimeresurus stejnegeri*	2.82%
13	Zinc metalloproteinase-disintegrin HV1	Unigene635_Cp	*Trimeresurus purpureomaculatus*	2.81%
14	group III snake venom metalloproteinase	E9KJZ5	*Echis ocellatus*	2.80%
15	Zinc metalloproteinase-disintegrin-like TSV-DM	Q2LD49	*Trimeresurus stejnegeri*	2.35%
16	Zinc metalloproteinase homolog/disintegrin	CL288.contig3_Ta	*Trimeresurus albolabris*	2.24%
17	Zinc metalloproteinase-disintegrin stejnihagin-A	CL92.contig5_Ta	*Trimeresurus albolabris*	1.67%
18	Snake venom metalloproteinase 5	J3S831	*Crotalus adamanteus*	1.19%
19	Zinc metalloproteinase-disintegrin-like batroxstatin-3	C5H5D4	*Bothrops atrox*	0.99%
**Snaclecs**				**19.43%**
1	Snaclec stejaggregin-B subunit beta-1	Q71RQ9	*Trimeresurus stejnegeri*	5.03%
2	Snaclec coagulation factor IX/factor X-binding protein subunit A	Q71RR4	*Trimeresurus stejnegeri*	4.90%
3	Mucrocetin subunit alpha	Unigene86_Ta	*Trimeresurus albolabris*	3.13%
4	Snaclec stejaggregin-B subunit alpha	Q71RQ7	*Trimeresurus stejnegeri*	2.88%
5	C-type lectin TsL	Q9YGP1	*Trimeresurus stejnegeri*	2.29%
6	C-type lectin Cal	P21963	*Crotalus atrox*	1.19%
**Snake venom serine proteases (SVSP)**				**14.27%**
1	Alpha-fibrinogenase albofibrase	P0CJ41	*Trimeresurus albolabris*	5.32%
2	Thrombin-like enzyme ancrod	P26324	*Calloselasma rhodostoma*	2.83%
3	Thrombin-like enzyme halystase	P81176	*Gloydius blomhoffii*	2.35%
4	Snake venom serine protease serpentokallikrein-2	Q9DG84	*Protobothrops mucrosquamatus*	2.13%
5	Snake venom serine protease KN13	Q71QH6	*Trimeresurus stejnegeri*	1.64%
**Phospholipases A_2_**				**5.40%**
1	Phospholipase A_2_	A0A0H3U232	*Trimeresurus sabahi*	4.27%
2	Basic phospholipase A_2_ homolog	P22640	*Protobothrops mucrosquamatus*	1.13%
**Disintegrins**				**5.26%**
1	Disintegrin ussuristatin-1	Q7LZI5	*Gloydius ussuriensis*	2.67%
2	Disintegrin trigramin-gamma	P62383	*Trimeresurus gramineus*	2.59%
**Cysteine-rich secretory proteins (CRiSP)**				**4.31%**
1	Cysteine-rich venom protein	P60623	*Trimeresurus stejnegeri*	3.84%
2	Cysteine rich secretory protein	T2HP25	*Protobothrops flavoviridis*	0.47%
**L-amino acid oxidase (LAAO)**				**0.99%**
1	L-amino acid oxidase	CL43.contig1_Ta	*Trimeresurus albolabris*	0.99%
**Phosphodiesterases (PDE)**				**0.37%**
1	Phosphodiesterase 1	CL2883.contig1_Cp	*Trimeresurus* *purpureomaculatus*	0.29%
2	Venom phosphodiesterase 1	J3SEZ3	*Crotalus adamanteus*	0.08%
**5′-nucleotidase (5′NUC)**				**0.15%**
1	Snake venom 5′-nucleotidase	CL554.contig1_Ta	*Trimeresurus albolabris*	0.15%
**Cellular proteins**				**1.42%**
1	Endonuclease domain-containing 1 protein-like	Unigene20352_Ec	*Echis carinatus*	0.63%
2	Endonuclease domain-containing 1 protein-like	Unigene20352_Ec	*Echis carinatus*	0.41%
3	Endonuclease domain-containing 1 protein-like CPACP	CL3153.contig1_Cp	*Trimeresurus* *purpureomaculatus*	0.29%
4	Glutaminyl-peptide cyclotransferase	Q90YA8	*Gloydius blomhoffii*	0.08%

^a,b,c^ Protein annotation, accession numbers and the corresponding species were derived from databases based on the best homology. ^b^ Protein codes with suffix “_CP,” “_Ta,” “_CrT” and “_Ec” were derived from the in-house database containing RNAseq specific for *Trimeresurus purpureomaculatus*, *Trimeresurus albolabris*, *Calloselasma rhodostoma* and *Echis carinatus*, respectively, available in Supplementary Table S2. ^d^ Protein abundance was interpreted as the percentage of total venom proteins.

**Table 2:** Efficacy of Green Pit Viper Antivenom (GPVAV) in neutralizing the toxic effects of *Trimeresurus nebularis* venom.

**Lethality**	***i.v.* LD_50_^a^ (µg/g)**	**Challenge Dose**	**ED_50_^b^ (µL)**	**ER_50_^c^ (mg/mL)**	**Potency, P ^d^ (mg/mL)**	**Normalized Potency, n-P ^e^ (mg/g)**
	2.00 (1.61–2.48)	5 LD_50_	100.00	2.00 (1.61–2.48)	1.6	79.2
**Procoagulant activity**	**MCD ^f^ (µg/mL)**	**Challenge Dose**	**ED ^g^ (µL)**	**ER ^h^ (mg/mL)**		
	150.0 ± 6.0	2 MCD	13.2 ± 0.5	4.6 ± 0.2	NA	NA
**Hemorrhagic activity**	**MHD ^i^ (µg)**	**Challenge Dose**	**ED_50_^j^ (µL)**	**ER_50_^k^ (mg/mL)**		
	1.67 ± 0.15	2 MHD	0.95 ± 0.13	3.52 ± 0.3	NA	NA

LD_50_: Median lethal dose; ED_50_: Median effective dose; ER_50_: Median effective ratio; MCD: Minimal clotting dose; MHD: Minimal hemorrhagic dose; ED: Effective dose; ER: Effective ratio; NA: Not applicable. Antivenom (GPVAV) protein concentration: 20.2 mg/mL [54]. ^a^ Median lethal dose was defined as the dose of venom (µg/mL) at which 50% of mice dead. ^b^ Median effective dose was defined as the dose of antivenom (µL) at which 50% of mice survived. ^c^ Median effective ratio was defined as the ratio of venom (mg) to the volume does of antivenom (mL) at which 50% of mice survived. ^d^ Potency, P was defined as the neutralization potency of the antivenom (mg/mL) at which the amount of venom (mg) was completely neutralized per unit volume of antivenom (mL). ^e^ Normalized P, n-P was defined as the neutralization potency of the antivenom (mg/g) at which the amount of venom (mg) completely neutralized per unit amount of antivenom protein (g). ^f^ Minimal clotting dose was defined as the dose of venom (µg/mL) required to cause clotting in 5 min. ^g^ Effective dose was defined as the dose of antivenom (µL antivenom) that was capable of prolonging the clotting time of challenge dose to 3 times. ^h^ Effective ratio was defined as the amount of venom neutralized per unit volume of antivenom (mg venom/mL antivenom) at which the clotting time of challenge dose was prolonged by 3 times. ^i^ Minimal hemorrhagic dose was defined as the amount of venom (µg) that induced a skin hemorrhagic lesion of 10 mm diameter. ^j^ Median effective dose was defined as the dose of antivenom (µL) at which the venom hemorrhagic activity was reduced by 50%. ^k^ Median effective ratio was defined as the amount of venom neutralized per unit volume of antivenom (mg venom/mL antivenom) at which the venom hemorrhagic activity was reduced by 50%.

## Supplementary Materials

The following are available online at http://www.mdpi.com/2072-6651/11/2/95/s1, Table S1: LC-MS/MS analysis for *Trimeresurus nebularis* venom proteome; Table S2: Sequence data of *de novo* transcriptomics applied in LC-MS/MS.
